# Neurofeedback learning modifies the incidence rate of alpha spindles, but not their duration and amplitude

**DOI:** 10.1038/s41598-017-04012-0

**Published:** 2017-06-19

**Authors:** Alexei Ossadtchi, Tatiana Shamaeva, Elizaveta Okorokova, Victoria Moiseeva, Mikhail A. Lebedev

**Affiliations:** 1 Center for Cognition and Decision Making, National Research University Higher School of Economics, Moscow, Russia; 2 Laboratory of Control of Complex Systems, Institute of Problems of Mechanical Engineering, Russian Academy of Sciences, St. Petersburg, Russia; 3 Pavlov Department of Physiology, Institute of Experimental Medicine, Russian Academy of Medical Sciences, St. Petersburg, Russia; 4 Department of Neurobiology, Duke University, Durham, North Carolina, United States of America

## Abstract

Although the first experiments on alpha-neurofeedback date back nearly six decades ago, when Joseph Kamiya reported successful operant conditioning of alpha-rhythm in humans, the effectiveness of this paradigm in various experimental and clinical settings is still a matter of debate. Here, we investigated the changes in EEG patterns during a continuously administered neurofeedback of P4 alpha activity. Two days of neurofeedback training were sufficient for a significant increase in the alpha power to occur. A detailed analysis of these EEG changes showed that the alpha power rose because of an increase in the incidence rate of alpha episodes, whereas the amplitude and the duration of alpha oscillations remained unchanged. These findings suggest that neurofeedback facilitates volitional control of alpha activity onset, but alpha episodes themselves appear to be maintained automatically with no volitional control – a property overlooked by previous studies that employed continuous alpha-power neurofeedback. We propose that future research on alpha neurofeedback should explore reinforcement schedules based on detection of onsets and offsets of alpha waves, and employ these statistics for exploration and quantification of neurofeedback induced effects.

## Introduction

Neurofeedback is a form of biofeedback, which allows subjects to observe and ultimately gain volitional control over their own brain activity. Neurofeedback settings involve recording of neural activity, extraction of neural features of interest, transformation of these features, and feeding the resulting signal back to the subject via one of sensory modalities: visual, auditory or tactile. Under these conditions, subjects learn to volitionally modify their brain activity, which they are normally unaware of.

One of the first neurofeedback experiments was conducted in the late 1960’s by Joe Kamiya^1, 2^. His basic neurofeedback design consisted of two stages. During the first stage, called the training session, subjects appreciated their own mental state (high/low alpha) using the feedback. During the second stage, they trained to enter the target (high alpha) state upon an acoustic prompt. Kamiya reported that after the training session 80% of the subjects managed to successfully classify their mental state and initiate it upon the prompt^3^. After learning the task, subjects reported mental states reflecting relaxation, “letting go” and pleasant feelings associated with cortical alpha activity.

A few years later, Barry Sterman demonstrated operant conditioning of cortical oscillations in cats. He studied the so-called sensorimotor (SMR) 12–15 Hz rhythm, which normally occurs in the motor cortex when an animal is in a state of alert immobility, often before attacking the prey. In Sterman’s study, cats learned to enter the SMR state following a conditioning stimulus. Additionally, the animals learned to produce the SMR even without any stimulus if food reinforcement was given each time they spontaneously entered the SMR state^4^. The same cats that underwent neurofeedback training were later used in an epilepsy study, and it was unexpectedly found that they were significantly less prone to seizures evoked by the epileptogenic fuel component than the cats that did not have the SMR training^5^. Thus, these experiments of Sterman and his colleagues revealed a potential of neurofeedback as a treatment for epilepsy^6–8^.

Despite these spectacular early findings, the field of neurofeedback remained underestimated and barely alive until the mid 1990s, when technological advances, including reasonably priced and efficient computerized EEG diagnostic equipment, brought impetus to EEG research, in general, and EEG-based neurofeedback, in particular. Since then, the number of studies has been growing on various aspects of neurofeedback in both laboratory and clinical settings^9^.

In addition to treatment of epilepsy, neurofeedback has been employed as a non-pharmacological therapy for a broad range of neurological conditions, such as ADHD^10–13^, depression^14, 15^ and autism spectrum disorder^16, 17^. Furthermore, neurofeedback therapy has been shown to improve sleep quality^18, 19^, mood^20^ and cognitive function and even enhance performance of sports and arts professionals^21–23^. Finally, a new and increasingly popular line of research employed neurofeedback as a key component of brain-computer interfaces (BCIs), systems that connect the brain to computers in order to assist and rehabilitate communication and motor functions to patients suffering from different degrees of paralysis caused by injuries and diseases of the brain and the spinal cord^24^. Properly chosen neurofeedback improves and enriches BCI operations^25, 26^.

The rapid revival of neurofeedback research including the use of various recording^27–33^, signal processing and source-localization techniques^34–36^, however, came along with a number of skeptical concerns about the validity of this approach. Conflicting results on neurofeedback effectiveness, as well as the absence of standardized neurofeedback protocols^37^ hinder our understanding of neurofeedback: what EEG features to use, where in the brain to record, and how to process EEG features in experimental studies and clinical applications.

Several recent methodological papers^23, 37–39^ outline the requirements for any state-of-the-art neurofeedback experiment. The temporal specificity of the reinforcement is one of the major concerns raised in these studies. Since neurofeedback is a reinforcement learning process^40, 41^, it is important that the reinforcement is delivered at the right time and that it triggers the desired brain adaptation. A reinforcement signal that arrives too late may not be properly matched with the desired activity pattern and, as a result, may impair or even prevent learning^42^. The choice of a relevant learning metrics for neurofeedback presentation has been a subject of long-lasting debate since the early experiments of Kamiya^43–45^. Typical neurofeedback protocols employ either discrete (percent-time) or continuous (integrated) schemes of reinforcement^38^. Another technique, called the Z-score neurofeedback, allows a generalized threshold-neurofeedback presentation based on a population distribution^46^ of the EEG parameter used. Other common considerations include the choice of sensory modality^47, 48^, complexity of reinforcement^49^, contingency of reinforcement^50, 51^, secondary reinforcement as an additional motivation factor^42^, and the shaping process for adjusting the feedback throughout an experiment^52^.

**Figure 1:**
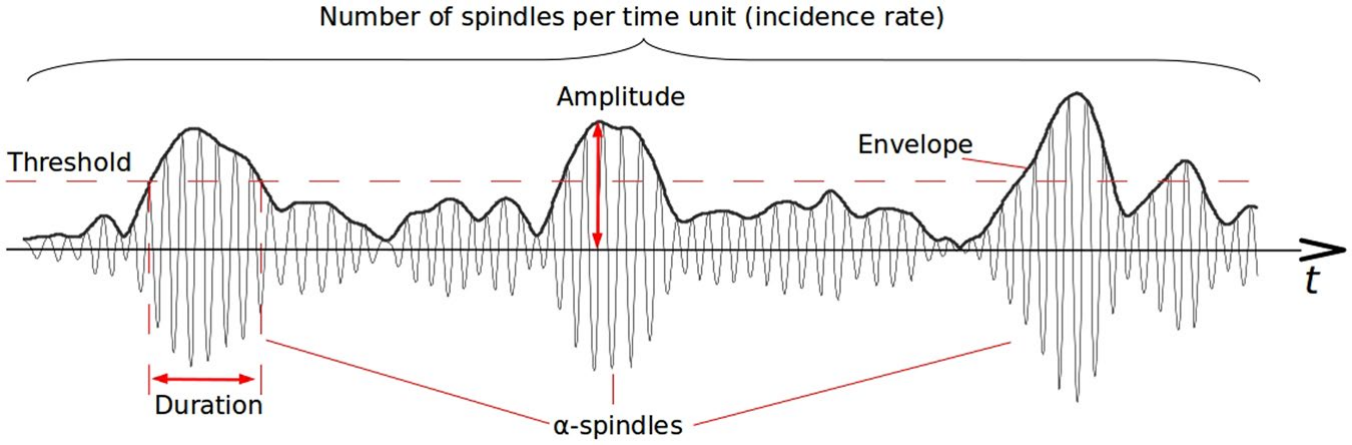
A representative EEG trace filtered in the 8–12 Hz band. Due to the inherent non-stationarity of the EEG time series, the average increase of the alpha-band power can be explained by changes in the duration of alpha spindles, the peak amplitude and the incidence rate of such spindles.

Simple continuous measures of EEG rhythms, which are commonly employed to generate neurofeedback, may obscure important details of the dynamical patterns contained in complex and non-stationary EEG signals. For example, changes in continuously monitored power of an EEG spectral band (Fig. 1) may be caused by changes in any of the three parameters that we employed in this study to quantify EEG patterns: spindle duration, amplitude and incidence rate. Accordingly, if EEG band power is used to generate the feedback, it is unclear which of the parameters the subjects can volitionally control. In order to achieve the optimal neurofeedback performance we need a more detailed knowledge of EEG patterns and their susceptibility to volition.

In this paper, we investigated the detailed spatiotemporal characteristics of EEGs using a traditional paradigm, where subjects received continuous neurofeedback based on their occipito-parietal (P4) alpha-rhythm power and were instructed to increase it. Neurofeedback training continued for two consecutive days in each subject and resulted in clear enhancements of alpha activity. Our analysis showed that, although the neurofeedback was based on a traditional continuous representation of alpha power, the underlying mechanism for the neurofeedback-induced EEG changes was best characterized as discrete. We propose that, taken one step further, this result warrants investigation into discrete EEG features as an approach to improve neurofeedback efficiency and explore and quantify the neurofeedback-induced effects.

## Methods

### Participants and settings

Eighteen healthy right-handed adults (12 females and 6 males) participated in the alpha neurofeedback training. The experiment was conducted in accordance with the ethical standards of the 1964 Declaration of Helsinki. All participants gave written informed consent prior to the experiment. The study was approved by the ethics committee of the St.-Petersburg State University.

The participants were randomly split into equally-sized experimental and control groups. The subjects in the experimental group received visual feedback that represented their EEG activity. Subjects in the control group received sham feedback based on prerecorded EEGs taken from a different subject. The participants from both groups received identical instructions from the same experimenter and adhered to the same experimental protocol.

The experiment was conducted in an electrically shielded room. Subjects sat in a comfortable chair in front of a conventional 14–inch LCD monitor with a 60 Hz refresh rate. The brightness settings were kept identical throughout the entire experiment and corresponded to the default (middle) position of the brightness slider.

### EEG recording and experimental procedure

**Figure 2:**
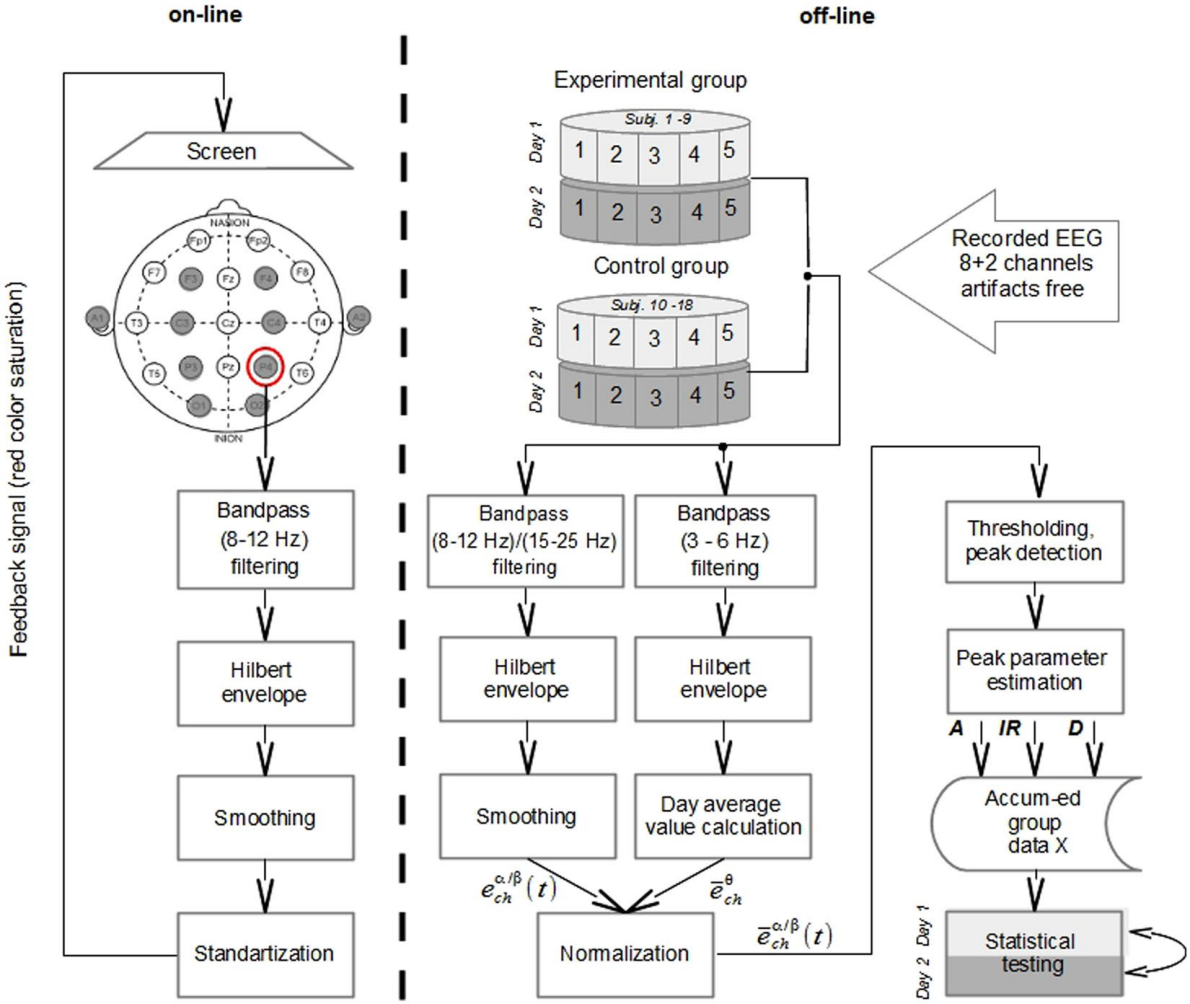
Schematic representation of the experimental procedure.

The left panel of Fig. 2 shows the real-time signal processing steps. We recorded EEG from eight channels (F1, F2, C3, C4, P3, P4, O1, O2) with passive Ag-Cl electrodes connected to Mitsar-21 amplifiers. The signals ground was set at Afz, and digitally linked ears were used as reference. Electrode impedance at each site was below 10 kΩ and sampling rate was 500 Hz.

The feedback signal was computed based on the EEG signal *x*(*t*) recorded from the P4 electrode placed over the right parietal region (marked by a circle in Fig. 2). This signal was band-pass filtered in the 8–12 Hz range, using the 4-th order Butterworth IIR filter to obtain the narrow-band process *x* ^*α*^(*t*). To calculate the instantaneous power *P*_*α*_(*t*) of the band-passed EEG we applied the Hilbert transform^53^ that produced the complex-valued analytic signal ${z}^{\alpha }(t)={x}^{\alpha }(t)+j{\hat{x}}^{\alpha }(t)$, and then measured the envelope ${e}^{\alpha }(t)=|{z}^{\alpha }(t)|$ and the instantaneous power ${p}_{\alpha }(t)=|{z}^{\alpha }(t{)|}^{2}$. Figure 1 illustrates the envelope and its relationship to the waveform of the narrow-band process. To calculate the feedback signal *P*_*α*_(*t*) we then smoothed the instantaneous power values *P*_*α*_(*t*) over the window of duration *T*_*w*_ = 200 ms as ${P}_{\alpha }(t)={\int }_{-{T}_{w}}^{0}{p}_{\alpha }(t-\tau )d\tau$. We used the mean *m*_*BL*_ and the standard deviation *σ*_*BL*_ of the feedback signal *P*_*α*_(*t*) calculated over the baseline interval of duration *T*_*b*_ to obtain the standardized feedback signal ${\tilde{P}}_{\alpha }(t)=\frac{{P}_{\alpha }(t)-{m}_{BL}}{{\sigma }_{BL}}$. This standardized value was then encoded as the intensity of the screen red color and presented to the subject. The resulting delay between the electrode signal and the change in screen color was in the range of 250–350 ms and was caused by filtering, smoothing and the delays induced by the hardware and computer operating system. This delay is within the range of acceptable feedback latency values as reported in ref. 38. The subjects were instructed to increase the red color intensity while sitting as still as possible and keeping their eyes open.

Each experimental session consisted of five 120-s segments (*T*_*p*_ = 120 s), separated by 30-s breaks (*T*_*r*_ = 30 s). Prior to each daily experimental session, a *T*_*b*_ = 60 s long baseline EEG recording was conducted to obtain the statistical data needed for standardization of alpha feedback to each individual’s baseline level. The experiment was conducted for two consecutive days in each subject, with one experimental session per day. The two days of training provided us the opportunity to analyze the effects of neurofeedback during the same day and across days and at the same time guaranteed that all the subjects could be consistently tested. As our subsequent analysis indicated the P4 site was not always the peak-alpha site and was used for neurofeedback signal calculation in all subjects for consistency.

### Offline processing

Prior to any further processing, we utilized the InfoMax ICA procedure to remove ocular artifacts. Due to the limited number of channels (N = 8), we removed no more than two ICA components that captured ocular artifacts. We then processed the EEG data collected during the two experimental sessions (one per day) for each subject (Fig. 2, right). Consistent with previous observations on the daily variability of EEG recordings due to differences in electrode placement, skin-electrode contact, electrode conductivity, cerebral blood flow and electromyographic interference^54^, we observed that alpha power fluctuated from the first to the second recording day. To compensate for these fluctuations, in the analyses where we compared the first day to the second day data, as it is formalized in (1), we normalized the daily time series *p*_*α*_(*t*) by the average magnitude of the theta-band $\overline{{p}_{\theta }}$. The latter band could be used for such calibration because it was affected by recordings instability factors in the same way.

1 $${Q}^{\alpha }(t)=\frac{{p}_{\alpha }(t)}{\overline{{p}_{\theta }}},$$

Where *p*_*α*_(*t*) is the instantaneous envelope samples of the alpha-band (8–12 Hz) at time instance *t*, and $\overline{{p}_{\theta }}$ is the theta-band (3–7 Hz) envelope averaged across all sessions for the same day.

#### Alpha-band power changes

We first explored the neurofeedback-related changes in *Q* ^*α*^(*t*) using the P4 channel data. The analysis was performed for the experimental and control groups. We used paired permutation test^55^ to assess the significance of the change in the average P4 *Q* ^*α*^(*t*) observed over the two training days. The test was applied separately to the experimental and control groups.

Next, to examined the spatial specificity of the neurofeedback-induced changes, we performed the same tests on the data from the other seven recording sites, besides P4. To represent these results we employed the topographic maps of log_10_ of the uncorrected *p*-values of the null hypothesis of no difference in the mean alpha-band *Q* ^*α*^(*t*) between the two days of training. To correct for multiple comparisons, we used Benjamini-Hochberg procedure^56^ for each topographic map individually. We used *p*-value threshold corresponding to *q* = 0.1 false discovery rate (FDR) level. On the topoplots, the electrodes whose *p*–values were below the threshold (and thus significant) are pointed to by the black arrows with the corresponding *p*-value shown in brackets. To examine the changes in the beta rhythm, we used the *Q* ^*β*^(*t*)-ratio (defined similarly to *Q* ^*α*^(*t*) but using the beta-band (15–25 Hz) filtered signal in the numerator of equation 1). We did not run this analysis for the theta-band data because the average power in this frequency range was used as a normalizing factor for the *Q*-ratio.

#### Alpha band signal morphology

Alpha band oscillations occur in the form of transient spindles. These patterns were described using three parameters, all contributing to the average alpha power: the duration of the alpha spindles, their mean amplitude and their incidence rate. An increase in any of these parameters would lead to an increase of the average alpha-band power (see Fig. 1). However, from the physiological point of view, each of these parameters reflects a unique functional characteristic of the underlying neural activity.

To assess how each of these three parameters was affected by the neurofeedback training, we performed the analysis outlined in Fig. 2. Using the trace of P4 *Q* ^*α*^(*t*), we detected alpha-spindles as the envelope spindles that exceeded the threshold set at twice the median value of P4 *Q* ^*α*^(*t*) computed over the entire period of training (two days). The duration of a spindle was calculated as the number of samples the envelope signal spends above the threshold and the spindle amplitude as the maximum value achieved within this segment.

We then determined the amplitude, spindle duration and incidence rate parameters for the alpha spindles. A paired permutation test was employed to compare these values between the first and the second days of training. This analysis was conducted separately for experimental and control groups. To examine the spatial specificity of the observed effects we performed the same analyses for the remaining EEG channels and used the 0.1 FDR level to mask significant *p*–values in the scenario of 8 multiple comparisons in each map.

We have also examined the daily trends of alpha-spindle parameters as a function of the training segment index (1–5, first day; 6–10, second day) for each subject in the experimental and control groups. The trend was quantified as correlation coefficient between the parameter of interest (average power, spindles incidence rate, spindles amplitude, duration) and training segment index. Correlation coefficients were calculated for each day separately and then averaged. We then explored the between-group differences in these correlation coefficients using non-parametric Wilcoxon rank-sum test.

## Results

In the experimental group, we found a significant increase in the average alpha power per training segment *Q* ^*α*^(*t*), $\bar{\rho }=0.69,p < 0.005$. In the control group, no significant change was observed ($\bar{\rho }=0.15,p > 0.43$). Thus, the increase in P4 alpha power was due to the neurofeedback and not to any other possible factors involved, such as subjects’ adaptation to the experimental routine.

**Figure 3:**
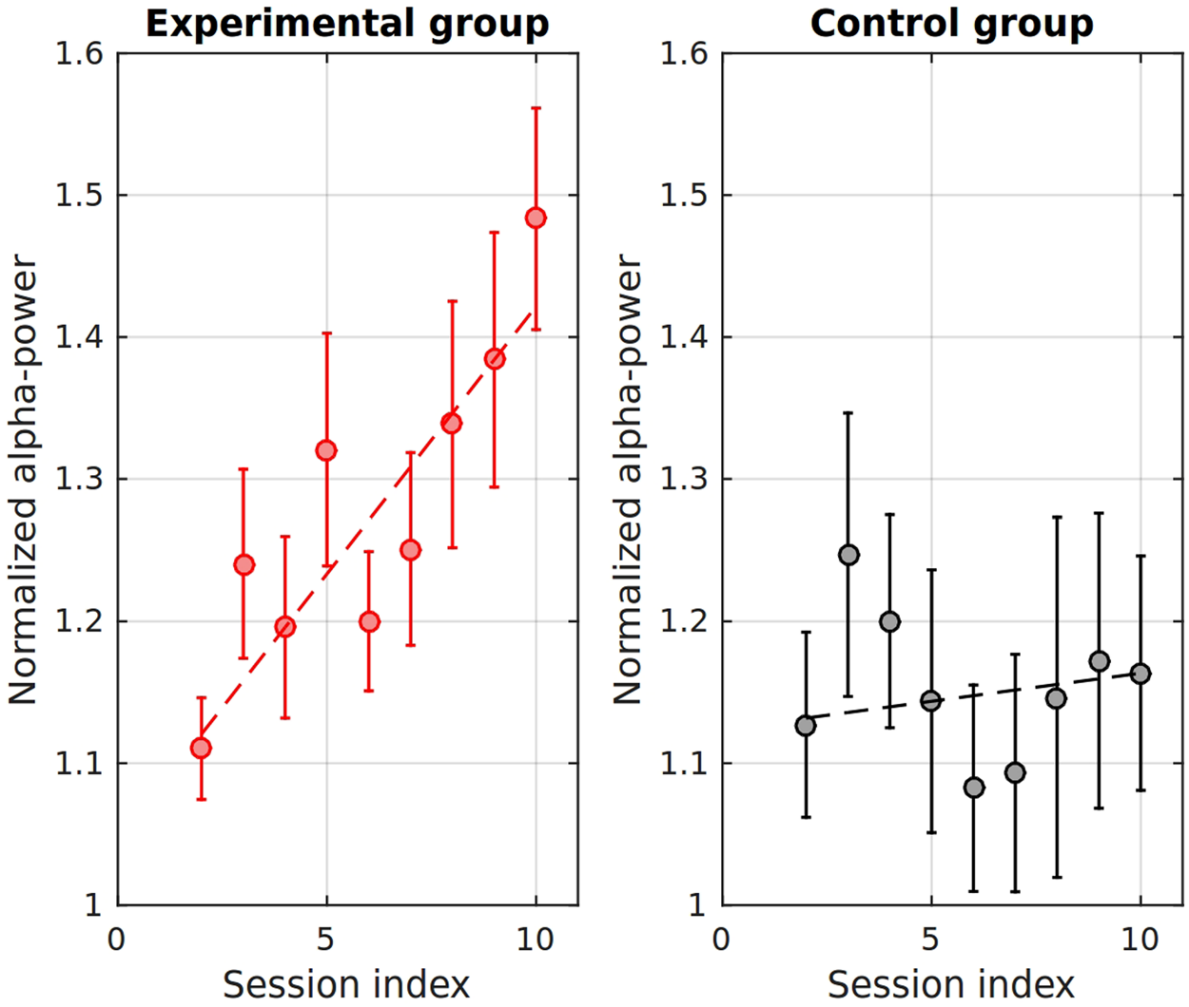
Average P4-alpha power dynamics in the experimental and the control groups during the two days of training. The points on the graph represent the mean alpha power during each 2-minute neurofeedback session relative to the first session of the first day. Since the first session of the first day was used as a reference point and is omitted from the graphs (baseline = 1), the effect of each individual 2-minute session can be estimated as a ratio of the observed value to that from the previous session.

Figure 3 shows the scatter plots of the average P4 *Q* ^*α*^(*t*) ratio calculated for each session in the experimental (left panel) versus control (right panel) groups. To facilitate comparison between subjects, each value was divided by the average P4 *Q* ^*α*^(*t*) from the first session (hence not shown in the plot). The values on the y-axis reflect the magnitude of the average Q-metric in the consecutive segments for the first (points 2–5) and second (points 6–10) days of training. The lines on the plots show the average trends across segments and allow to appreciate the effect of each individual training segment.

**Figure 4:**
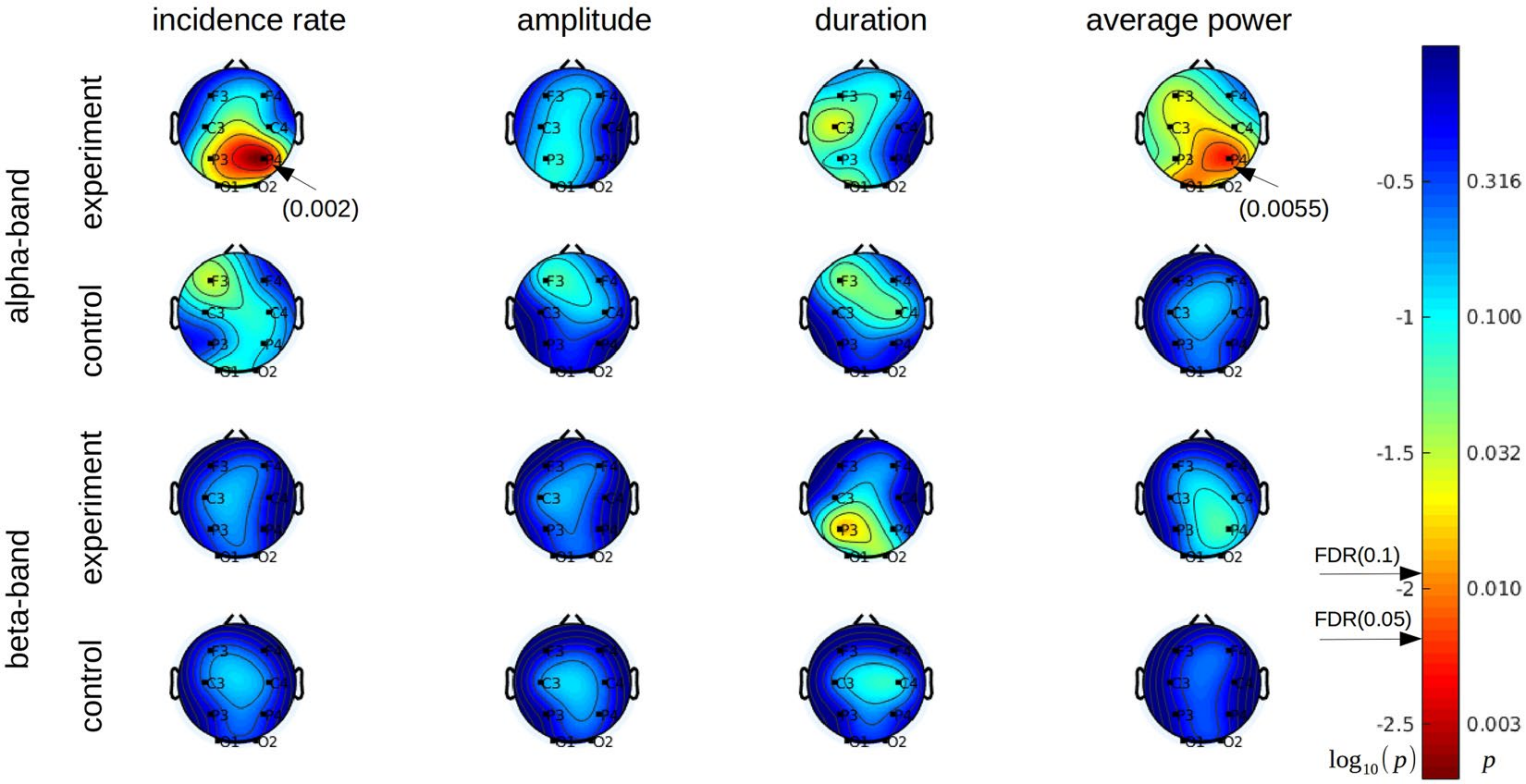
Statistical analysis of *Q* ^*α*^(*t*) waveform morphology in terms of the incidence rate of alpha spindles (first column), their duration (second column), amplitude (third column) and average power (fourth column). The electrodes exhibiting significant changes are pointed by the arrows with the corresponding *p*–values specified in brackets. A linear colormap encodes the log10 *p*-values obtained from the permutation test of no difference between days 1 and 2. Non-logarithmic *p*-values are also shown on the outer side of the colorbar. Beta band changes are included in the analysis as a control for frequency band specificity. Significant differences that pass the FDR (0.1) correction for multiple comparisons are observed only for the spindles incidence rate parameter at P4 (p = 0.002) and for the average power at the same electrode (p = 0.0055). These differences correspond to a greater mean value observed on day 2, as compared to day 1. Note that changes in the incidence rate parameter are more spatially specific and better correspond to the goal of neurofeedback training of enhancing alpha rhythm power at P4 than the average power changes (top row, rightmost column).

Next, we investigated the *Q* ^*α*^(*t*) waveform composition, namely the alpha spindles incidence rate, their average duration and amplitude (Fig. 1). Figure 4 shows the topographic plots reflecting the changes in these parameters between the first and the second day of neurofeedback training. The colors represent the log_10_ of the *p*-values, obtained using a two-sided permutation test with the null hypothesis of no difference in the parameters between day 1 and day 2. The same analysis was conducted for the higher EEG band (beta: 15–25 Hz). All significant and marginally significant differences were positive, i.e. day 2 > day 1.

In summary, Fig. 4 shows the following results:

1. As expected, the statistically significant increase of alpha-band power on the second day (top-row, rightmost column) occurred only in the experimental group and was most pronounced at P4 (*p* = 0.0055) which also passes the FDR 0.05 threshold. This effect was not observed in the beta band. At the same time, the effect was not spatially specific to P4 only because small but marginally significant changes were found in the central and frontal left regions (around C3 and F3). Possibly, neurofeedback training of the P4 alpha band led to a concomitant increase of alpha activity in other alpha-specific circuits, including the left sensory-motor regions, where high alpha activity (including the mu-rhythm) is considered to be an indicator of a motor idling state^57^.
2. As it can be seen in the leftmost column of the topomaps, the incidence rate parameter exhibited the most prominent spatially specific (i.e. with a clear peak over P4) and significant (*p* = 0.002) neurofeedback-induced change that passes the FDR = 0.05 threshold calculated for each map individually. The change in the other two parameters (duration and amplitude), for both experimental and control groups, did not exceed the significance threshold of 0.05 and was above the FDR = 0.1 threshold.
3. No significant increase of the incidence rate was observed in the non-targeted by neurofeedback beta band.
4. At the same time, we observed a marginally significant change in the beta-band burst duration at P3 in the experimental group and the duration parameter of alpha-spindles at C3. These small changes possibly reflecte alterations of the sensorimotor rythm (SMR) that accompanied the main effects of neurofeedback.
5. In the control group, a marginally significant increase occurred in the F3 alpha-spindle incidence rate parameter in the control group that could possibly relate to frontal asymmetry associated with stress and emotional state in response to incongruent feedback^58, 59^.

**Figure 5:**
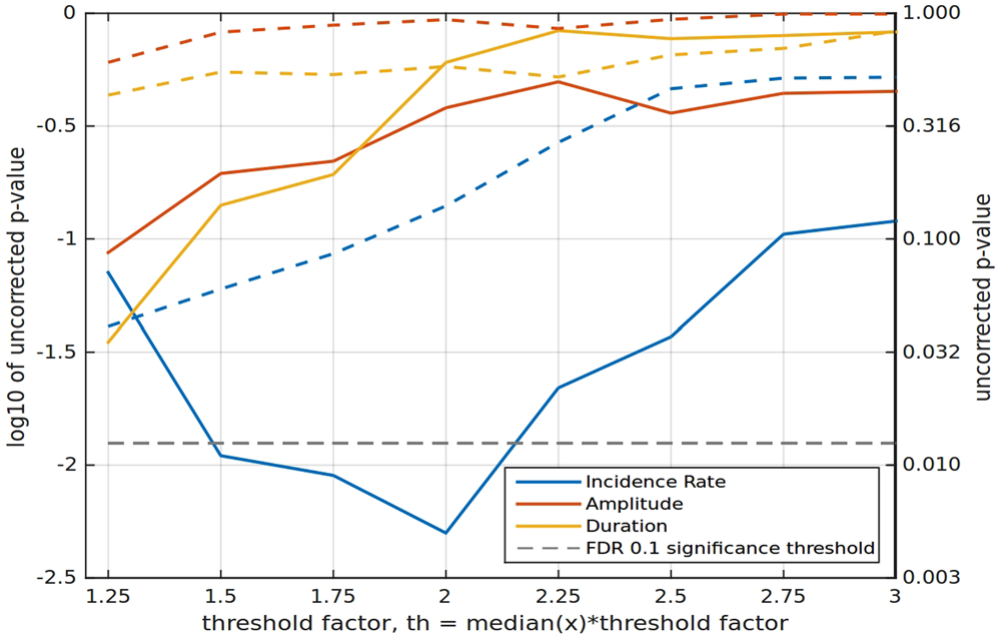
The effect of varying the spindle detection threshold. The *p*–value for the three parameters (spindle amplitude, incidence rate and duration) calculated from P4 electrode data for different threshold values is shown. The dashed lines correspond to the sham feedback group and the solid lines to the experimental group. The observed significance of alpha spindles incidence rate increase persists for a large range of threshold values.

As an additional verification, we tested different thresholds for alpha-spindle detection. We found that the results persisted for a large range of threshold values. Figure 5 depicts the *p*–values for the three parameters of the EEG at P4 (spindle amplitude, incidence rate and duration) calculated for different threshold values. The dashed lines correspond to the control group and the solid lines to the experimental group. Clearly, alpha-spindle incidence rate enhancement persisted for different threshold values, so this effect was robust to changes in the spindle-detection algorithm parameters.

**Figure 6:**
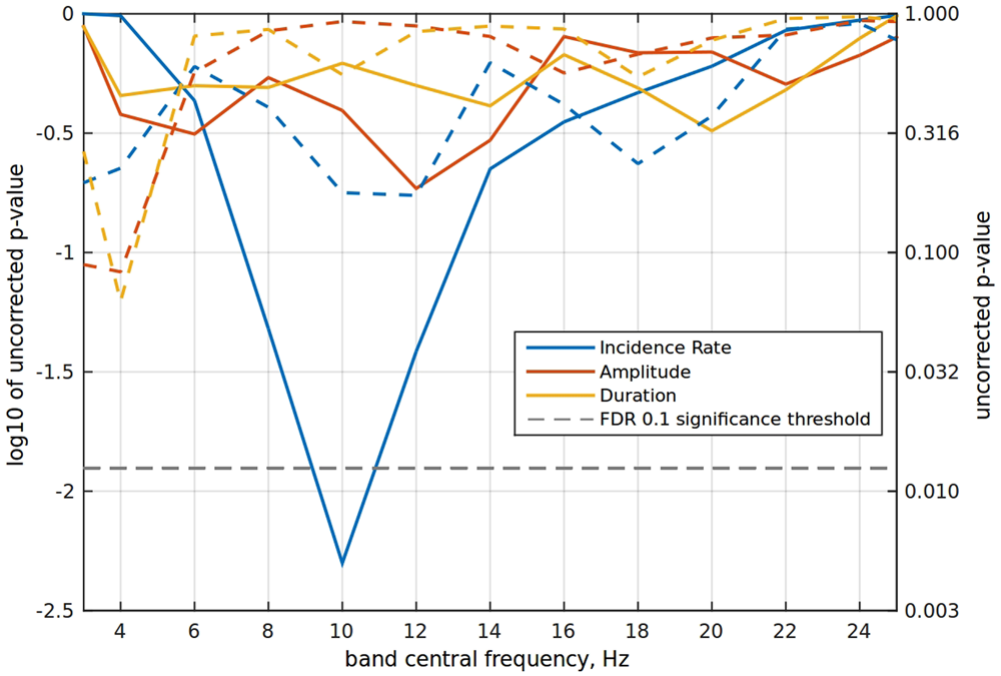
Band specificity study. ﻿The plot shows dependence of the main effect’s *p*– value on the band. Solid lines correspond to the experimental group and the dashed lines to the control group. The significant difference between days in the incidence rate parameter is observed only in the alpha-band and only in the experimental group.

For this purpose, we analyzed the P4 EEG signal in both the experimental and the control datasets using a sliding window (Fig. 6).﻿ The analysis utilized the same procedures as for the alpha band, and covered the range of frequencies from 3 to 25 Hz. This analysis showed that the incidence rate changed significantly only for the alpha band, but not for the other tested frequencies; and only in the experimental group.

**Figure 7:**
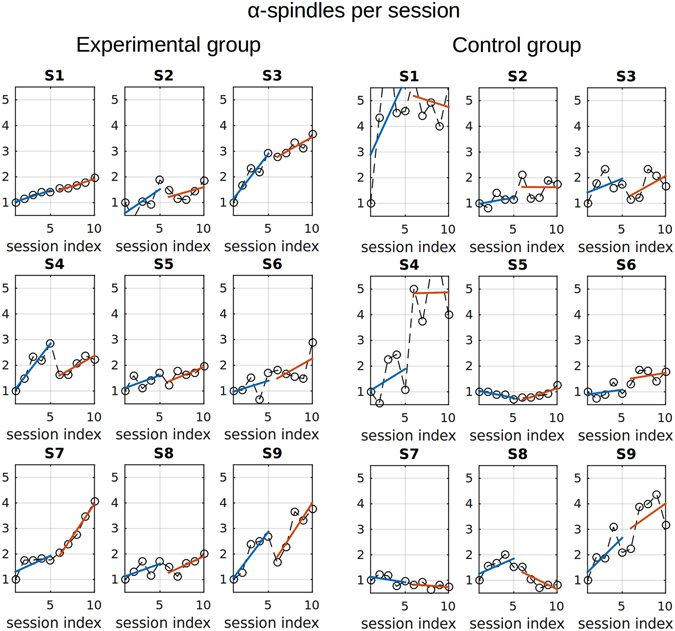
The dynamics of alpha-spindles incidence rate as a function of the training segment index (1–10) for all subjects (n = 9 experimental, n = 9 control) groups. The first five segments correspond to the the first day and the last five to the second day of training. For each subject, we normalized the spindles count by the number of events observed in the 1st segment (first day). The top panel corresponds to the experimental group and the bottom panel represents the data for the control group. All the subjects from the experimental group exhibit reliable positive correlation of within day training segment index. For quantification of the between group effects see Figure 8.

**Figure 8:**
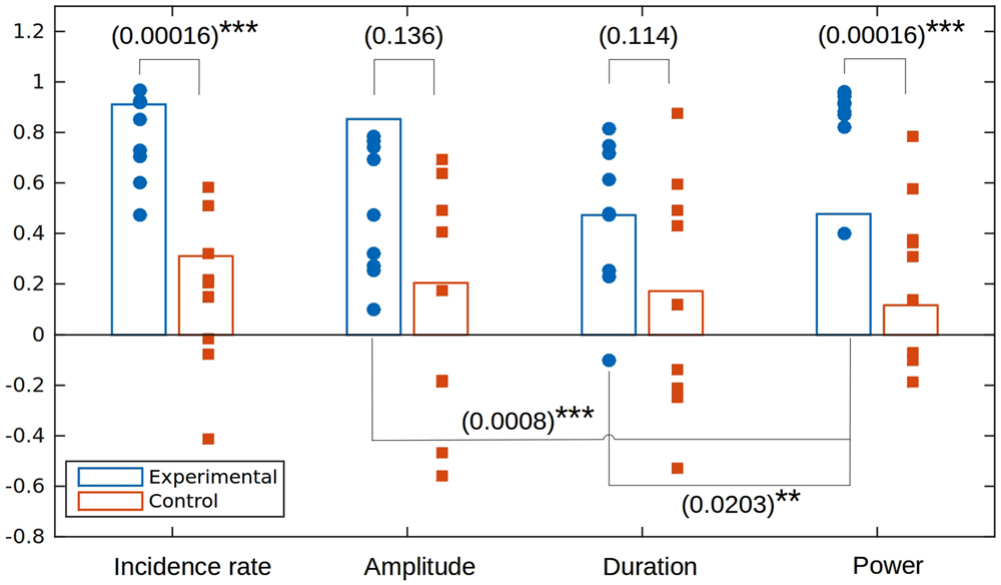
Summary of the results of the correlation analysis for different alpha-activity parameters for the experimental and control groups. For each subject, we plotted the points representing daily correlation coefficient for each of the four parameters of alpha activity: average power, spindles incidence rate, spindles amplitude and duration. Additionally, the bar plots represent average correlation coefficients for each group (blue for the experimental group; orange for the control group). Between group comparison shows that the experimental group data demonstrate high average correlation coefficients (*p* < 0.0001) for the power and spindles incidence rate parameters. No significant correlations are observed for the control group. Only the average power and spindles incidence rate features demonstrate highly significant differences between the experimental and control groups (Wilcoxon rank-sum test, *p* = 0.00016). No statistically significant differences are observed between the experimental and the control groups for spindle amplitude (*p* = 0.136) and spindle duration (*p* = 0.114) features. Only within the experimental group we observe a highly significant positive difference between the studied correlation coefficients for the spindle incidence rate and spindle amplitude parameter (*p* = 0.0008) as well as the spindle duration parameter (*p* = 0.0203).

Next, we analyzed changes in EEG patterns as a function of training segment for each day separately. The top and bottom panels of Fig. 7 show alpha-spindle incidence rate as a function of training segment for the experimental and the control groups, correspondingly. In these graphs, the first five points correspond to the five segments of the first day, and the last five points correspond to the five segments of the second day. For all subjects in the experimental group, alpha-spindle incidence rate had a tendency to increase during each training day. Mathematically, we expressed this tendency as a positive correlation between the segment number (index) and the spindle incidence rate. We did not observe such a tendency for the control group (bottom panel of Fig. 7), where different subjects exhibited different inconsistent direction of changes. Figure 8 summarizes the results of the correlation analysis for different alpha-activity parameters for the experimental and control groups. Here again, the correlation coefficient was calculated for the relationship between the segment number (1–5; each point represents an average for two recording days) and the parameter of interest. For each subject, we plotted the points representing daily correlation coefficient for each of the four parameters of alpha activity: average power, spindles incidence rate, spindles amplitude and duration. Additionally, the bar plots represent average correlation coefficients for each group (blue for the experimental group; orange for the control group). The statistical analysis for the two groups showed statistically significant positive correlations (with the training segment index) (*p* < 0.0001) in the experimental group only for the power and spindles incidence rate, but not for the spindle amplitude and duration. No significant correlations were found for any of the parameters in the control group. For the statistical comparison of the two groups, average power and spindles incidence rate differed significantly (Wilcoxon rank-sum test, *p* = 0.00016) whereas the other parameters did not. Also, within the experimental group the correlation coefficient for the spindle incidence rate was highly significantly different from the one for the amplitude (*p* = 0.0008) and significantly different from the one for spindle duration (*p* = 0.00203), which, once again, shows that the incidence rate was the major parameter affected by the neurofeedback training.

## Discussion

This study revealed novel properties of alpha rhythm pattern changes induced by neurofeedback training. We examined three characteristics of alpha activity: (1) the number of alpha spindles per unit of time (incidence rate), (2) the average spindle duration, and (3) the amplitude of alpha spindles, and found that only the rate of alpha spindles changed significantly during P4 alpha neurofeedback training. Seven out of nine (78%) subjects responded to training if judged by the between day comparison and all subjects responded to training if quantified by the within day alpha power increase (see Figures 7 and 8).

The significant and steady increase of the average P4 alpha-power with the number of training segments was clearly present in the experimental group and absent in the control group (see Fig. 3). This result was expected from the previous literature on alpha neurofeedback (for example, see studies on alpha-band power training)^21^. Our detailed analysis of the EEG patterns did not show a statistically significant increase in the amplitude and duration of alpha spindles. Instead, it turned out that the observed increase in alpha-band power was explainable solely by the increased incidence rate of alpha spindles. Therefore, what our subjects actually learned during the neurofeedback training was the ability to more easily transition to the brain state hallmarked by a pronounced alpha activity. Overall, our findings indicate that the analysis of neurofeedback-induced changes in EEG patterns should extend beyond the classical signal-processing metrics. These metrics are suitable for the analysis of stationary signals, but they fail to reflect the variability of non-stationary signal features. Taken one step further, these results favor the idea of the development of discrete form of reinforcement to improve alpha neurofeedback.

Our result can be considered from a historic perspective of neurofeedback training. From the very early experiments of Kamiya^1, 2^, it was common to measure neurofeedback learning in terms of two indices: the percent-time and integrated amplitude. The percent time metrics, also confusingly referred to as discrete neurofeedback, is calculated as the percent of data samples in the feedback signal that exceed a predefined amplitude threshold. The integrated alpha, on the other hand, is a continuous variable, measured as a time integral of the feedback signal, or, equivalently, the average amplitude of the signal. Both metrics could be used as a post processing tool or as a trigger for reinforcement in the online mode.

In 1976, Hardt and Kamiya^43^, sparked a controversy regarding the usage of percent time index. They pointed out that most of the successful studies on alpha neurofeedback utilized continuous features of the feedback signal (also see Ancoli)^60^. According to Hardt and Kamiya, percent-time alpha measure is restricted to detecting when the state is on or off, but does not demonstrate minor, yet important, changes in EEG activity. This failure of percent-time metric to capture the signal dynamics may discourage learners whose volitional EEG modifications are discarded by the threshold algorithm. Additionally, this metric may fail to distinguish good learners whose EEG modifications substantially exceed the threshold from average learners who barely exceed the threshold.

Lanski *et al*.^61^, in their comment to Hardt and Kamia^43^, proposed that the percent-time metrics, despite its failure to capture small changes in the feedback signal, allows for better and more natural alpha spindle detection, which is a special feature of the alpha rhythm. They also claimed that threshold based techniques allow better tracking of the spindle frequency. By contrast, alpha integration method requires a rather long time frame to perform computation: several minutes in Hardt and Kamiya study and, generally, around 300–500 ms in modern settings. Such prolonged processing delays reinforcement, masks fast signal dynamics and, as a result, significantly hinders learning.

Travis *et al*.^44^ investigated alpha enhancement during eyes-closed and eyes-open neurofeedback using both metrics. They found that learning with eyes-closed versus eyes-open was more effective with discrete-type neurofeedback, while learning with eyes-open gained from continuous feedback. In an attempt to resolve the controversy with the neurofeedback metrics, Dempster and Vernon^45^ compared three measures: integrated alpha amplitude, percent time, and a combination between the two. In the within-session analysis, all three patterns produced the same alpha-enhancing result. Their between-session analysis revealed a change in amplitude, but not the other two metrics, which Travis *et al*.^44^ found consistent with the work of Hardt and Kamiya^43^.

Our approach to analyzing alpha-activity is different from the two described metrics commonly used in the literature. We, for the first time, investigated discrete structural characteristics of EEG patterns. Even the “percent-time” metric known as a discrete measure, is, in fact, a continuous variable, representing the percent of time, the EEG signal exceeded the threshold over some immediate past time interval. Different from this approach, we isolated onsets and offsets of individual alpha spindles as discrete events, which allowed us to measure two parameters that could affect the overall prominence of alpha activity: incidence rate and duration of alpha episodes. We also calculated spindle amplitude that should not be confused with integrated amplitude, since the amplitude in our connotation is the characteristic of an individual alpha spindle, whereas the integrated amplitude refers to the averaging technique that does not discriminate between the spindle and non-spindle episodes.

Our findings clarify the results obtained with the previous metrics of neurofeedback. Our experimental conditions were similar to the previous studies: we utilized a continuous neurofeedback based on the integrated amplitude of alpha oscillations. In agreement with the literature, training with this neurofeedback resulted in a steady increase in the average alpha power, which is consistent with the results reported by Travis *et al*.^44^ using the integrated amplitude metrics. However, when we examined EEG patterns that underlied this enhancement in alpha activity, we found discrete modifications in the signal (i.e. increased frequency of spindle onsets) instead of generalized, continuous changes, such as increases in alpha amplitude and longer alpha episodes. Importantly, these discrete changes occurred even though we did not reinforce them specifically.

We did not find significant changes in the average duration and amplitude of alpha spindles over the two days of training. We also observed significantly stronger statistical association with the training segment index for incidence rate of alpha spindles than for their amplitude and duration. This result supports the interpretation that alpha spindles represent automatically generated cortical patterns that cannot be volitionally modified once they are started, even when aided by a neurofeedback. This suggests that alpha duration and amplitude are either stable within a physiological range or depend on the factors not directly related to the neurofeedback intervention. This line of reasoning is consistent with the early skepticism regarding EEG neurofeedback measures^38, 43, 48^.

Several observations suggest that the increase in alpha spindles incidence rate was a true effect of neurofeedback. First, this effect was observed only in the experimental group and was absent in the control group, which rules out adaptation to the experimental conditions as a possible explanation. Second, the effect was band specific: only alpha activity was affected, see Figs 4 and 7, indicating frequency specificity of the significant changes. Third, the effect was spatially specific: the strongest enhancement of spindle incidence rate occurred, as expected, at P4, the recording location from which neurofeedback was derived (Fig. 4). Furthermore, our results suggest that traditional metrics may fail to detect some neurofeedback-induced changes and properly reflect the spatial specificity of neurofeedback induced effects. For example, the conventional alpha power metric (rightmost column of Fig. 4) has poor spatial resolution; in addition to the expected changes for P4 location, it shows marginally significant change for F3 and C3. These observations warrant further studies of the neurofeedback induced effects with the use of advanced, yet physiologically plausible, metrics. In addition to our approach for the neurofeedback aimed at up-regulation of alpha-activity, recent studies demonstrated that training aimed at down-regulating occipital alpha oscillations led to a significant increase in the Long Range Temporal Correlations (LRTC)^62, 63^. Notably, in the former study^62^ an inverse U-relationship between LRTC and alpha oscillations amplitude was observed, where the upper point of the U-shape corresponded to an optimal excitation-inhibition balance. These studies add to our conclusion that the effects of continuous neurofeedback are not limited to merely changes in EEG power at different scalp locations.

Even though we used a traditional continuous neurofeedback in these experiments, our subjects modulated the occurrences of alpha-spindle onsets – a discrete characteristic – while spindle duration and spindle amplitude did not change. This response to neurofeedback is better described as learning to transition more frequently to a discrete alpha-state than achieving a gradual change in alpha power. Based on these findings, it is reasonable to suggest that the neurofeedback-susceptible discrete metric that we discovered offline could by itself constitute a new and more efficient type of neurofeedback in future experiments. Tentatively, subjects could be given an indicator each time they enter the target EEG state. Such discrete event-based neurofeedback could be also delivered in a continuous way if a floating average of spindle rate is provided to the subject instead of individual spindle onsets. Irrespective of implementation details, the underlying computation would be very different from the previous continuous types of neurofeedback. In addition to exploiting alpha rhythm, discrete structure of other EEG rhythms could be utilized to enrich neurofeedback. Previously, a mixture of continuous characteristics of different EEG rhythms was used to produce a two-dimensional spatial neurofeedback^64^. Such an experiment could plausibly benefit from using discrete EEG parameters. Using discrete EEG parameters obtained from different electrodes simultaneously is another possibility. We propose that all these ideas be explored in the future.

The ultimate test of the efficiency of discrete neurofeedback should be conducted in real-time setting. The future experiments should resolve a number of questions concerning the implementation of spindle onset-based metrics of alpha activity. Despite the plausible benefits of informing the user about spindle onset with a discrete signal, it still has to be determined whether such discrete neurofeedback should be used alone or, alternatively, in combination with continuous types of neurofeedback. Completely replacing the continuous neurofeedback with its discrete version may fail to achieve the goal of alpha enhancement because this feedback would not be sufficiently smooth^65, 66^.

Yet another problem related to discrete neurofeedback setting is the choice of the threshold for detecting alpha spindle onsets. Proper setting of the threshold is essential to facilitate neurofeedback-based training^48^ and the observed effect of spatially specific spindles incidence rate increase is observed for some specific (yet broad) range of threshold values (see Fig. 5). An algorithm with a very low threshold may confuse true alpha spindles with different brain states, whereas a very high threshold may discard true low-amplitude alpha oscillations triggered by the subject’s volition, and, as a result, frustrate the subject. In this paper we derived the threshold from the median values for the data collected over two days. Such statistics is not available in a typical on-line setting. Additionally, a fixed threshold is not suitable for tracking learning-related signal changes and making appropriate adjustments to the spindle detection algorithm. Additionally, as noticed by Hardt and Kamiya^43^, discrete metrics fail to provide information about the signal characteristics below or above the threshold. This problem could be addressed, for example, by setting multiple time-varying thresholds that increase the information content of the feedback signal.

With these considerations in mind, the future experiments should compare the efficiency of alpha neurofeedback training for continuous, discrete and mixed feedback, in order to reveal the type of setting needed to achieve fast and long-lasting alpha neurofeedback plasticity, and produce the desired cognitive gains.

### Limitations

Our neurofeedback study has several limitations. The first limitation is related to a relatively small sample size (9 participants in each group). A larger size would be desirable for the follow-up studies to fully capture the effect of neurofeedback intervention. Additionally, we only tested the effect of neurofeedback during the two days of training and did not examine the same subjects several days or months after the intervention. This will be done in our future work.

In this study, we investigated the patterns of alpha feedback signal, but we did not employ neurofeedback to train the other commonly used frequency bands, such as beta, theta or SMR. The dynamics of the spindles incidence rate, their duration and amplitude could be different for these other frequency ranges or other training conditions, e.g. eyes-closed. It would be also of interest to examine the effect of the delay between the detected EEG patterns and neurofeedback on the subjects’ ability to control these patterns. If the feedback is not rapid enough subjects may not be able to track pattern changes efficiently. For example a 250–300 ms delay of this study could possibly limit the ability to detect alpha-spindles onsets and offsets, hindering the control.

Finally, we still have very limited understanding of how discrete-type reinforcement could be different from continuous reinforcement in terms of its cognitive and clinical effects. It might happen that each reinforcement type enhances different neural processes, essential for some interventions, but not the others. All these gaps in our knowledge should be answered by further investigation. Future work should clarify the pros and cons of discrete, mixed and continuous feedback, determine the methods for inducing sustained plasticity and optimize these techniques so that desired cognitive gains could be achieved.

## References

[1] Kamiya, J. Conscious control of brain waves. *Psychology Today* (1968).

[2] Kamiya, J. Operant control of the EEG alpha rhythm and some of its reported effects on consciousness. *Altered states of consciousness*. *New York: Wiley* **1069** (1969).

[3] Nowlis DP, Kamiya J (1970). The control of electroencephalographic alpha rhythms through auditory feedback and the associated mental activity. Psychophysiology.

[4] Wyrwicka W, Sterman MB (1968). Instrumental conditioning of sensorimotor cortex EEG spindles in the waking cat. Physiology & Behavior.

[5] Sterman M, LoPresti R, Fairchild M (2010). Electroencephalographic and behavioral studies of monomethyl hydrazine toxicity in the cat. Journal of Neurotherapy.

[6] Sterman M, Friar L (1972). Suppression of seizures in an epileptic following sensorimotor EEG feedback training. Electroencephalography and clinical neurophysiology.

[7] Sterman M, MacDonald L, Stone RK (1974). Biofeedback training of the sensorimotor electroencephalogram rhythm in man: effects on epilepsy. Epilepsia.

[8] Tan G (2009). Meta-analysis of EEG biofeedback in treating epilepsy. Clinical EEG and Neuroscience.

[9] Evans, J. R. & Abarbanel, A. *Introduction to quantitative EEG and neurofeedback* (Elsevier, 1999).

[10] Zuberer A, Brandeis D, Drechsler R (2015). Are treatment effects of neurofeedback training in children with adhd related to the successful regulation of brain activity? A review on the learning of regulation of brain activity and a contribution to the discussion on specificity. Frontiers in human neuroscience.

[11] Vernon D, Frick A, Gruzelier J (2004). Neurofeedback as a treatment for adhd: A methodological review with implications for future research. Journal of Neurotherapy.

[12] Arns M, de Ridder S, Strehl U, Breteler M, Coenen A (2009). Efficacy of neurofeedback treatment in adhd: the effects on inattention, impulsivity and hyperactivity: a meta-analysis. Clinical EEG and neuroscience.

[13] Lofthouse N, Arnold LE, Hurt E (2012). Current status of neurofeedback for attention-deficit/hyperactivity disorder. Current psychiatry reports.

[14] Hammond DC (2005). Neurofeedback treatment of depression and anxiety. Journal of Adult Development.

[15] Linden D (2012). Real-time self-regulation of emotion networks in patients with depression. PloS one.

[16] Lofthouse, N., Hendren, R., Hurt, E., Arnold, L. E. & Butter, E. A review of complementary and alternative treatments for autism spectrum disorders. *Autism research and treatment* **2012** (2012). 10.1155/2012/870391 PMC3515887 23243505

[17] Coben R, Linden M, Myers TE (2010). Neurofeedback for autistic spectrum disorder: a review of the literature. Applied psychophysiology and biofeedback.

[18] Hoedlmoser K (2008). Instrumental conditioning of human sensorimotor rhythm (12–15 hz) and its impact on sleep as well as declarative learning. Sleep.

[19] Cortoos A, De Valck E, Arns M, Breteler MH, Cluydts R (2010). An exploratory study on the effects of tele-neurofeedback and tele-biofeedback on objective and subjective sleep in patients with primary insomnia. Applied psychophysiology and biofeedback.

[20] Raymond J, Varney C, Parkinson LA, Gruzelier JH (2005). The effects of alpha/theta neurofeedback on personality and mood. Cognitive brain research.

[21] Gruzelier JH (2014). EEG-neurofeedback for optimising performance. i: A review of cognitive and affective outcome in healthy participants. Neuroscience & Biobehavioral Reviews.

[22] Gruzelier JH (2014). EEG-neurofeedback for optimising performance. ii: creativity, the performing arts and ecological validity. Neuroscience & Biobehavioral Reviews.

[23] Gruzelier JH (2014). EEG-neurofeedback for optimising performance. iii: A review of methodological and theoretical considerations. Neuroscience & Biobehavioral Reviews.

[24] Birbaumer N, Murguialday AR, Cohen L (2008). Brain–computer interface in paralysis. Current opinion in neurology.

[25] Neuper, C. & Pfurtscheller, G. Neurofeedback training for bci control. In *Brain-Computer Interfaces*, 65–78 (Springer, 2009).

[26] Hwang H-J, Kwon K, Im C-H (2009). Neurofeedback-based motor imagery training for brain–computer interface (bci). Journal of neuroscience methods.

[27] Florin E, Bock E, Baillet S (2014). Targeted reinforcement of neural oscillatory activity with real-time neuroimaging feedback. Neuroimage.

[28] Caria A, Sitaram R, Birbaumer N (2012). Real-time fmri a tool for local brain regulation. The Neuroscientist.

[29] Lawrence EJ (2014). Self-regulation of the anterior insula: reinforcement learning using real-time fmri neurofeedback. Neuroimage.

[30] Ruiz S, Buyukturkoglu K, Rana M, Birbaumer N, Sitaram R (2014). Real-time fmri brain computer interfaces: self-regulation of single brain regions to networks. Biological psychology.

[31] Mihara M (2013). Near-infrared spectroscopy–mediated neurofeedback enhances efficacy of motor imagery–based training in poststroke victims a pilot study. Stroke.

[32] Kober SE (2014). Near-infrared spectroscopy based neurofeedback training increases specific motor imagery related cortical activation compared to sham feedback. Biological psychology.

[33] Zotev V, Phillips R, Yuan H, Misaki M, Bodurka J (2014). Self-regulation of human brain activity using simultaneous real-time fmri and EEG neurofeedback. NeuroImage.

[34] Cannon R (2006). EEG spectral-power and coherence: Loreta neurofeedback training in the anterior cingulate gyrus. Journal of Neurotherapy.

[35] Cannon R, Congedo M, Lubar J, Hutchens T (2009). Differentiating a network of executive attention: Loreta neurofeedback in anterior cingulate and dorsolateral prefrontal cortices. International Journal of Neuroscience.

[36] Koberda JL, Koberda P, Bienkiewicz AA, Moses A, Koberda L (2013). Pain management using 19-electrode z-score loreta neurofeedback. Journal of Neurotherapy.

[37] Thibault RT, Lifshitz M, Raz A (2016). The self-regulating brain and neurofeedback: Experimental science and clinical promise. cortex.

[38] Sherlin LH (2011). Neurofeedback and basic learning theory: implications for research and practice. Journal of Neurotherapy.

[39] Strehl U (2014). What learning theories can teach us in designing neurofeedback treatments. Frontiers in human neuroscience.

[40] Skinner BF (1958). Reinforcement today. American Psychologist.

[41] Caria A, Sitaram R, Veit R, Begliomini C, Birbaumer N (2010). Volitional control of anterior insula activity modulates the response to aversive stimuli. a real-time functional magnetic resonance imaging study. Biological psychiatry.

[42] Grice GR (1948). The relation of secondary reinforcement to delayed reward in visual discrimination learning. Journal of experimental psychology.

[43] Hardt JV, Kamiya J (1976). Conflicting results in EEG alpha feedback studies. Biofeedback and Self-regulation.

[44] Travis T, Kondo C, Knott J (1974). Parameters of eyes-closed alpha enhancement. Psychophysiology.

[45] Dempster T, Vernon D (2009). Identifying indices of learning for alpha neurofeedback training. Applied psychophysiology and biofeedback.

[46] Thatcher, R. W. & Lubar, J. F. (eds) *Z Score Neurofeedback- Clinical Applications* (Elsevier, Amsterdam, 20015).

[47] Hinterberger T (2004). A multimodal brain-based feedback and communication system. Experimental brain research.

[48] Vernon D (2009). Alpha neurofeedback training for performance enhancement: reviewing the methodology. Journal of neurotherapy.

[49] Pearce JM, Hall G (1978). Overshadowing the instrumental conditioning of a lever-press response by a more valid predictor of the reinforcer. Journal of Experimental Psychology: Animal Behavior Processes.

[50] Trowbridge MH, Cason H (1932). An experimental study of thorndike’s theory of learning. The Journal of General Psychology.

[51] Siniatchkin M, Kropp P, Gerber W-D (2000). Neurofeedbackâ€”the significance of reinforcement and the search for an appropriate strategy for the success of self-regulation. Applied psychophysiology and biofeedback.

[52] Sherlin LH, Larson NC, Sherlin RM (2013). Developing a performance brain training™ approach for baseball: A process analysis with descriptive data. Applied psychophysiology and biofeedback.

[53] Bracewell, R. The Fourier Transform and Its Applications (McGraw–Hill, 2000).

[54] Bazanova O, Vernon D (2014). Interpreting EEG alpha activity. Neuroscience & Biobehavioral Reviews.

[55] Nichols TE, Holmes AP (2002). Nonparametric permutation tests for functional neuroimaging: a primer with examples. Human brain mapping.

[56] Benjamini Y, Hochberg Y (1995). Controlling the false discovery rate: a practical and powerful approach to multiple testing. Journal of the Royal Statistical Society, Series B.

[57] Pineda JA (2005). The functional significance of mu rhythms: translating “seeing” and “hearing” into “doing”. Brain Research Reviews.

[58] Quaedflieg, C. *et al*. The validity of individual frontal alpha asymmetry EEG neurofeedback. *Social cognitive and affective neuroscience* nsv090 (2015). 10.1093/scan/nsv090 PMC4692315 26163671

[59] Stewart JL, Coan JA, Towers DN, Allen JJ (2014). Resting and task-elicited prefrontal EEG alpha asymmetry in depression: Support for the capability model. Psychophysiology.

[60] Ancoli S, Kamiya J (1978). Methodological issues in alpha biofeedback training. Biofeedback and Self-regulation.

[61] Lansky P, Bohdanecký Z, Indra M, Radil-Weiss T (1979). Alpha detection. Biofeedback and Self-regulation.

[62] Ros, T. *et al*. Neurofeedback tunes scale-free dynamics in spontaneous brain activity. *Cerebral Cortex* 1–12 (2016). 10.1093/cercor/bhw285 27620975

[63] Zhigalov, A., Kaplan, A. & Palva, J. M. Modulation of critical brain dynamics using closed-loop neurofeedback stimulation. *Clinical Neurophysiology* **127**, 2882–2889 (2016). 10.1016/j.clinph.2016.04.028 27256308

[64] Jonathan, R. W. & Dennis, J. M. Control of a two-dimensional movement signal by a noninvasive brain-computer interface in humans. *Proceedings of the National Academy of Sciences of the United States of America* **101**, 17849–17854 (2004). 10.1073/pnas.0403504101 PMC535103 15585584

[65] Bennett, S. *A history of control engineering, 1930–1955*. 47 (IET, 1993).

[66] Zeyda, F., Aranyi, G., Charles, F. & Cavazza, M. An empirical analysis of neurofeedback using pid control systems. In *Systems, Man, and Cybernetics (SMC), 2015 IEEE International Conference on*, 3197–3202 (2015).

